# A Comparison of Methods of Gut Microbiota Transplantation for Preclinical Studies

**DOI:** 10.3390/ijms241512005

**Published:** 2023-07-26

**Authors:** Jonas Mingaila, Alessandro Atzeni, Aurelijus Burokas

**Affiliations:** Department of Biological Models, Institute of Biochemistry, Life Sciences Center, Vilnius University, Sauletekio Ave. 7, LT-10257 Vilnius, Lithuania; jonas.mingaila@gmc.vu.lt

**Keywords:** fecal microbiota transplantation, 16S sequencing, 18S sequencing, gut microbiota, gut mycobiota, antibiotic

## Abstract

The experimental details reported in preclinical fecal microbiota transplantation (FMT) protocols are highly inconsistent, variable, and/or incomplete. We therefore evaluated FMT from a human donor to antibiotic-induced microbial-depleted mice by exploring the effects of six techniques based on antibiotic (AB) or antibiotic + antimycotic (AB + T) gut decontamination, different administration routes, and different dosing intervals on the gut microbial population, assessed using 16S and 18S sequencing. In addition, we explored the effectiveness of FMT in terms of inflammation, physiological, and behavioral outcomes. Our results showed that intrarectal FMT at low dosing intervals better preserved the donor’s gut bacterial community at genus level. Furthermore, we showed a lower abundance of several genera of fungi in animals treated with AB + T. In addition, we observed that AB + T gut decontamination followed by per os FMT, once every 3 days, affected behavioral parameters when compared to other FMT techniques. Accordingly, the same FMT groups that showed an association with some of the behavioral tests were also related to specific gut fungal genera, suggesting a possible mediation. Our findings may be useful for optimizing the practice of FMT and also in terms of donor microbiota preservation. This information may help to improve the reproducibility and reliability of FMT studies.

## 1. Introduction

The gut microbiota has recently emerged as an important therapeutic target for various health conditions, from gastrointestinal disease to neuropsychiatric disorders [1,2,3], and fecal microbiota transplantation (FMT) represents an important tool in this research field [4]. FMT consists of transferring the gut microbial community from a donor to a recipient, and it has been widely demonstrated that, clinically, this procedure can alleviate *Clostridium difficile* infection, but also several other diseases like irritable bowel syndrome, inflammatory bowel diseases, insulin resistance, multiple sclerosis, and idiopathic thrombocytopenic purpura [5,6]. Indeed, the challenging but also ambiguous results related to the therapeutic applications of FMT have led to the creation of clear recommendations and guidelines that are constantly being updated and improved [7]. These reports contain comprehensive information describing donor selection, fecal bacteria extraction, the type of administration, and doses [8].

Furthermore, the use of FMT has also increased preclinically as a powerful tool to elucidate the cause–effect relationship between microbiota and disease in animal models [9,10,11,12]. However, in contrast with clinical FMT protocols, there is a need for guidelines or recommendations on best practices and standardization for the preclinical use of FMT [13].

Mouse models are widely used in preclinical FMT experiments, especially when transferring fecal microbiota from human donors, because it is possible to “humanize” the microbiota of these animals by mimicking the composition and profile of the donor [14]. 

The use of germ-free (GF) mice is considered the gold standard for animal-based FMT studies. These animals do not host any resident microorganisms in their gut, and for this reason, they are often used as recipients in FMT models. However, some limitations in the use of GF mice are faced in terms of high costs and logistics [15]. In addition, it is important to mention other drawbacks related to the limited development of the immune system compared to conventionally raised animals [16], the effects on brain development [12], and the presence of intestinal disfunctions [17].

Therefore, antibiotic-induced microbial depletion results in a valid approach to avoiding the issues faced in the use of GF animals, allowing the normal colonization and correct development of the immune system [18]. Nevertheless, some studies have already highlighted consistent limitations, especially related to the composition, administration, duration, and dose of antibiotics, which can have detrimental effects on animal health [18], but also, from an experimental point of view, the introduction of extreme variability that can induce confounding results when inferring causality [13].

In addition, when conducting FMT procedures from human donors to animal models, it is important to consider that the gastrointestinal tract is also colonized by a large population of fungi, which can also play an important role in intestinal homeostasis and can affect the relationship between the donor and host in FMT preclinical procedures. But again, some important factors from this point of view are scarce in the literature [19].

Accordingly, the aim of this study was to address the gap in the literature by providing useful information regarding the best practice for FMT from human donors to antibiotic-induced microbial-depleted mice by exploring the effects of different techniques (antibiotic treatment, administration route, and duration) on gut bacterial and fungal populations, but also in terms of inflammation and behavioral outcomes, in order to finally detect which procedure produces better results.

## 2. Results

### 2.1. Behavioral and Physiological Characteristics of the Animals Included in This Study

In the current study, 34 C57BL/6JRj adult male mice were divided into six distinct FMT technique groups (A, B, C, D, E, and F), which differed from one another in terms of the type of gut decontamination treatment (AB, antibiotic cocktail; AB + T, antibiotic cocktail plus terbinafine), the route of FMT administration (PO, per os; IR, intrarectal), and the dosing intervals (times/day). At the end of the experiments, information regarding intestinal transit time, pellet humidity, and blood and behavioral parameters was collected.

The main characteristics of the six FMT groups and the differences in behavioral and physiological parameters are reported in Table 1.

Animals in group C showed a higher intestinal transit time than animals in group B and group D (261.2 ± 54.6 m vs. 197.7 ± 41.9 m and 195.6 ± 24.2 m).

Regarding behavioral parameters, animals in group B showed a higher total track length (4623.3 ± 565.2 cm) than animals in group A (3973.4 ± 407.0 cm); meanwhile, animals in group E showed a higher total track length (4996.4 ± 588.9 cm) than animals in group A (3973.4 ± 407.0 cm), group C (4299.1 ± 380.8 cm), and group D (4104.4 ± 323.6 cm).

The percentage of activity during the experiment was higher in group E (60.9 ± 3.9) compared to group A (55.8 ± 4.3). Velocity was higher in group E (12.1 ± 2.4 cm/s) compared to group A (9.2 ± 1.8 cm/s) and group C (9.3 ± 1.4).

### 2.2. Differences in Gut Bacterial Community Profiles between Human Donor and FMT Groups

We observed that the Chao1 alpha diversity index was higher in the human donor vs. FMT group B (*p* = 0.043), whereas the Shannon and Simpson indices were higher in the human donor vs. all FMT groups (Table 2).

**Figure 1:**
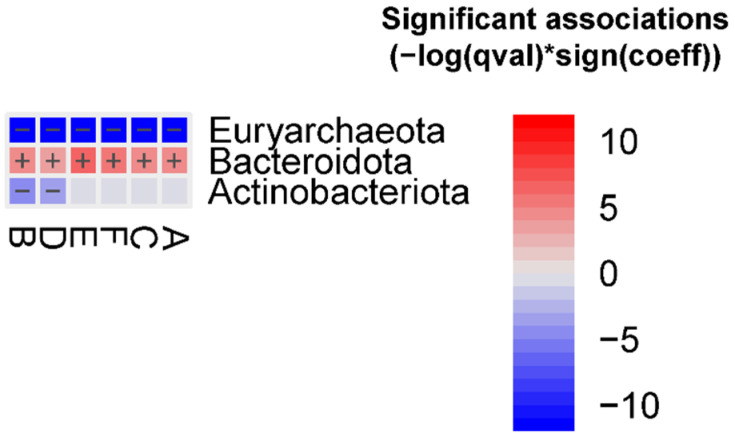
Significant bacterial phyla associated with the different FMT groups vs. the human donor (FDR < 0.05), detected using the MaAslin2 linear model. Detailed results are reported in the Supplementary Materials, Table S1.

The abundance of the Euryarchaeota phylum was decreased in all FMT groups, whereas the abundance of the Bacteroidota phylum was enriched in all FMT groups vs. the human donor. In addition, the Actinobacteriota phylum was decreased in groups B and D vs. the human donor (Figure 1).

At the genus level, we observed 252 differential abundant features between the human donor and different FMT groups (Table S2). Among them, 18 of 43 features were enriched in the human donor vs. group A; 20 of 48 features were enriched in the human donor vs. group B; 17 of 43 features were enriched in the human donor vs. group C; 19 of 47 features were enriched in the human donor vs. group D; 18 of 45 features were enriched in the human donor vs. group E; and 9 of 26 features were enriched in the human donor vs. group F.

### 2.3. Differences in Gut Bacterial Community Profiles across Different FMT Conditions

No differences in the Chao1 alpha diversity index were observed across the FMT groups. Animals in group F showed a lower Shannon index compared to animals in groups A (*p* = 0.022), C (*p* = 0.018), and E (*p* = 0.017). Animals in group F showed a lower Simpson index compared to animals in groups A (*p* = 0.044), B (*p* = 0.041), and E (*p* = 0.037) (Table 3). No differences in alpha diversity were observed between the different gut decontamination treatments, FMT administrations, and dosing intervals (Table S3).

No differences in the Aitchison distance were observed between the different FMT conditions (Table S5). 

**Figure 2:**
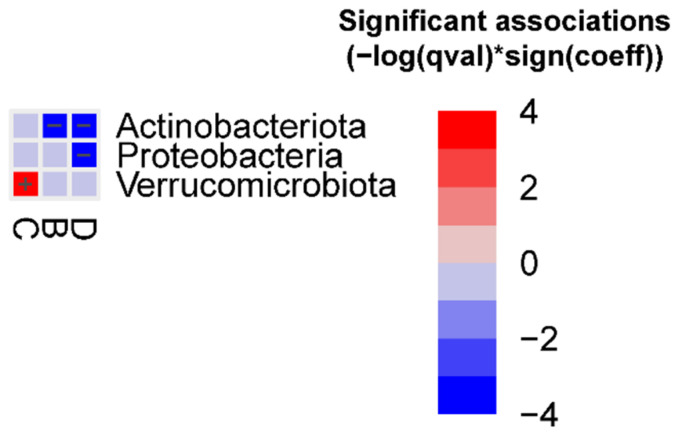
Significant bacterial phyla associated with the different FMT groups (FDR < 0.05) vs. group F, detected using the MaAslin2 linear model. Detailed results are reported in the Supplementary Materials, Table S4.

**Figure 3:**
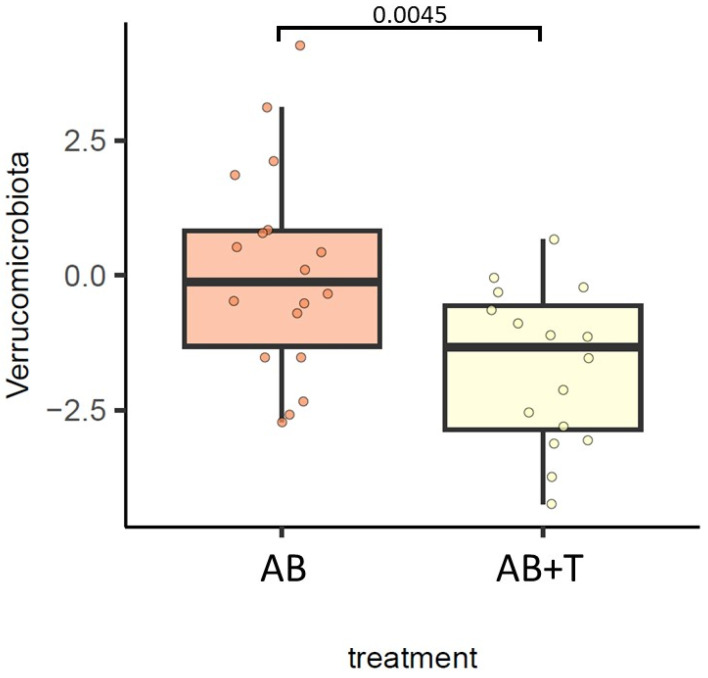
Differences in the gut bacterial community at the phylum level between gut decontamination treatments. The results of the generalized linear model are shown. FDR < 0.05 was deemed significant.

At the phylum level, we observed that Actinobacteriota was decreased in groups B and D vs. group F, Proteobacteria was decreased in group D vs. group F, and Verrucomicrobiota was increased in group C vs. group F (Figure 2). In addition, we observed that Verrucomicrobiota was enriched in animals treated with AB (FDR = 0.0045) (Figure 3). 

At the genus level, we did not observe any differential abundant features across the different FMT conditions.

### 2.4. Differences in Gut Fungal Community Profiles between Human Donor and FMT Groups

There were no differences in the calculated alpha diversity indices between the human donor and different FMT groups (Table S6).

At the phylum level, we did not observe any differential abundant features between the human donor and different FMT groups.

At the genus level, we observed that *Nakaseomyces Candida clade* was enriched in the human donor vs. groups A, B, and C; *Saccharomices* was enriched in the human donor vs. group A; *Funneliformis* was enriched in the human donor vs. groups A and C; and *Tremellales* was enriched in the human donor vs. group A (Table 4).

### 2.5. Differences in Gut Fungal Community Profiles across Different FMT Conditions

The Chao1, Shannon, and Simpson alpha diversity indices were lower in group B vs. group F (*p* = 0.014, *p* = 0.001, and *p* = 0.008, respectively). The Shannon and Simpson indices were lower in group B vs. group E (*p* = 0.014 and *p* = 0.037, respectively), and in group C vs. group E (*p* = 0.021 and *p* = 0.033, respectively) and group F (*p* = 0.002 and *p* = 0.007, respectively). Moreover, we observed a lower Shannon index in group D vs. group F (*p* = 0.044) (Table 5). Furthermore, the Shannon and Simpson indices were higher in animals exposed to the AB treatment (*p* = 0.005 and *p* = 0.013, respectively) (Table S7). No differences were observed between the different FMT administrations or dosing intervals.

We observed significant differences in the Aitchison distance between the different FMT groups (*p* = 0.002) and different gut decontamination treatments (*p* = 0.002) (Table 6). The multiple group comparison showed that the Aitchison distance was significantly higher in group F vs. group B (Bonferroni-adjusted *p*-value = 0.030) (Table S8).

**Figure 4:**
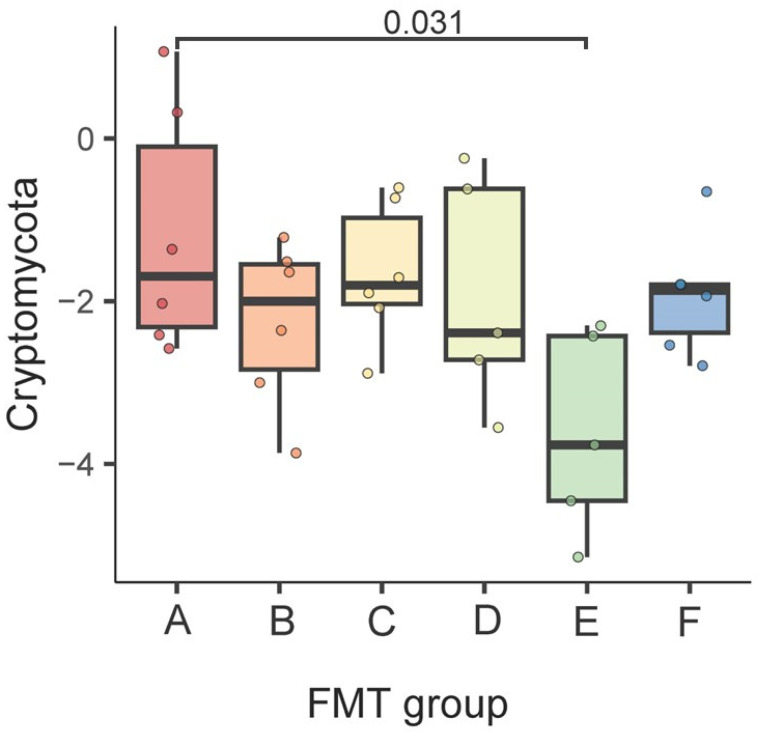
Differences in the gut fungal community at the phylum level between the FMT groups. The results of the generalized linear model are shown. FDR < 0.05 was deemed significant.

At the phylum level, we observed that Cryptomycota was enriched in group A vs. group E (FDR = 0.031) (Figure 4). 

The results of the differential abundance analysis at the genus level across the different FMT groups are shown in Table 7. *Symmetrospora* was enriched in group C vs. groups A (FDR = 0.010), B (FDR = 0.032), and F (FDR = 0.015); *Melanopsichium* was enriched in group A vs. groups C (FDR = 0.030) and D (FDR = 0.011); *Camptobasidiaceae* was enriched in group E vs. groups A (FDR = 0.017) and C (FDR = 0.036); *Cheilymenia* was enriched in group D vs. groups A (FDR = 0.009) and E (FDR = 0.029); *Nakaseomyces/Candida clade* and *Curvularia* were enriched in group D vs. group A (FDR = 0.046 and FDR = 0.048, respectively); *Tremellales* and *Tranzscheliella* were enriched in group E vs. group A (FDR = 0.010 and FDR = 0.046, respectively); *Paramicrosporidium* was enriched in group A vs. group E (FDR = 0.045); and *Kondoa* was enriched in group A vs. group F (FDR = 0.030).

In addition, animals treated with AB + T showed a larger number of enriched genera (nine) than decreased genera (five) when compared with animals treated with AB (Table 8).

### 2.6. Gut Microbiota Mediation in the Association between FMT Techniques and Different Behavioral Parameters

For the 16S dataset, the results of the differential abundance analysis at the genus level across the different FMT groups did not show associations with the FMT groups (group A vs. B; group E vs. A, C, and D) that showed relationships with the behavioral tests of interest reported in Table 1 (total track length, activity during the experiment, velocity).

On the other hand, according to the results shown in Table 7 for the 18S dataset, the fungal genera *Tremellales*, *Camptobasidiaceae*, *Paramicrosporidium*, and *Tranzscheliella* were associated with group E vs. group A; *Camptobasidiaceae* was associated with group E vs. group C; and *Cheilymenia* was associated with group E vs. group D. To test the potential mediation of the gut microbiota in the association between FMT techniques and behavioral tests of interest, the abundances of these genera were selected, and the association was tested against the total track length, activity during the experiment, and velocity. The results showed a positive association between the fungal genera *Tremellales*, *Camptobasidiaceae*, and *Tranzscheliella* and the behavioral parameters total track length and activity during the experiment (*p* = 0.006, *p* = 0.003, and *p* = 0.034, respectively), as well as a positive association between *Camptobasidiaceae* and velocity (*p* < 0.001) (Table 9).

## 3. Discussion

In the current study, we aimed to define the differences in terms of the gut bacterial and fungal communities in groups of animals exposed to different FMT techniques, and the possible implications for several parameters.

For all the FMT techniques considered in this study, we observed a significantly lower gut bacterial alpha diversity compared with the donor, highlighting the overall effects of gut bacterial decontamination. It has been observed that mice treated with an antibiotic cocktail of ampicillin, vancomycin, neomycin, and metronidazole, prior FMT via oral gavage, at a low dosage received effective gut decontamination and a successful engraftment of the donor microbiota [18]. Accordingly, we observed that lower dosing intervals reduced the number of differential abundant genera in comparison with the human donor. 

Comparing four different methods based on the FMT frequency over a period of 4 weeks (twice a week, once a week, two FMTs, one FMT), Wrzosek and colleagues showed that FMT administration twice a week for four weeks was too frequent and perturbed the stability of the gut microbial ecosystem, whereas FMT once a week allowed bacterial engraftment and a higher diversity [20]. Thus, we observed that animals exposed to less frequent FMT showed higher alpha diversity compared to animals exposed to more frequent FMT. On the other hand, it was observed that mice treated with a 3-week antibiotic regimen followed by 5-daily FMT showed a greater resemblance to the human donor microbiota compared with mice treated with a 3-day antibiotic regimen followed by 1-daily FMT [21]. However, when comparing different FMT frequencies separately, we did not observe any differences, suggesting that, perhaps, inter-group differences may be attributed to other FMT method-related factors. At present, the optimum FMT dosage to sustain the donor microbiota in the recipient is not clear [12], highlighting the lack of studies permitting answers to this question and the importance of our study from this point of view.

We observed that the gut microbiota of animals treated via intrarectal administration at a lower dosage showed a higher capacity to restore the donor’s microbiota and higher alpha diversity compared with the groups of animals exposed to FMT via oral gavage. It has been speculated that intrarectal administration is more effective than oral gavage, as the inoculum does not need to pass through different gastrointestinal barriers. However, the optimum choice of FMT administration remains a debated topic [13]. According to our results, the FMT techniques did not show effects on the outcomes of inflammation, such blood parameters, meaning it was not possible to determine which type of administration was less harmful to the animals.

We observed that the main difference in the gut microbial community across the study groups was related to the mycobiota (the population of fungi inhabiting the gut) identified through 18S sequencing. The importance of this portion of the gut microbial community to host homeostasis has already been highlighted, but there is a lack of studies identifying the best FMT approach to preserve it. In addition, it was observed that components of the gut mycobiota may contribute to disease recurrence in patients with *Clostridium difficile* infection treated with FMT [19]. It has been demonstrated that intestinal colonization with mucosal fungi increased the responsiveness of mice to social stimuli, highlighting the ability of gut fungi to modulate host behavior [22]. Accordingly, we observed that higher behavioral parameters were associated with a higher abundance of specific genera of fungi in animals treated with AB + T. Animals treated with AB + T and administered FMT PO once ×3 days showed a higher total track length and velocity than animals treated with AB, and animals treated with AB + T with larger FMT dosing intervals. Furthermore, the activity during the experiment was also higher in group E compared to animals treated with AB with larger FMT dosing intervals.

Exploring the potential mediation of the gut microbiota in the association between the FMT groups and behavioral tests, the main signal only came from the 18S dataset when comparing animals exposed to the AB + T treatment vs. animals exposed to the AB treatment. Accordingly, the same FMT groups that showed an association with some of the behavioral tests were also related to specific gut fungal genera.

In conclusion, FMT at low dosing intervals seems to be the best technique to better preserve donors’ gut bacterial population. However, it seems that the main signal that may help to elucidate the best FMT condition comes from the 18S dataset. Animals exposed to AB + T gut decontamination showed a lower abundance of several fungal genera. In addition, behavioral parameters were affected in animals exposed to AB + T gut decontamination, followed by per os FMT once ×3 days, in comparison with animals treated with AB, and animals treated with AB + T with larger FMT dosing intervals. Several fungal genera appear to mediate this association. This study helps to identify the best FMT procedure in mice, considering a number of different modalities not fully described in the literature, by providing useful information for more comprehensive guideline development. We also aimed to help in standardizing methods for FMT in animals and to improve preclinical studies. Our study presents some limitations, especially related to the small sample size and the inability to generalize our results to other routes. However, from this point of view, these limitations can be used as an advantage when making further improvements.

## 4. Methods

### 4.1. Animals

A total of 34 adult male inbred strain C57BL/6JRj mice, aged 8 weeks (Janvier Labs, France), were group-housed (6 groups) under a 12 h light–dark cycle, in controlled laboratory conditions with a temperature of 21 ± 1 °C and humidity of 55 ± 10% over the whole duration of the experiments. Standard rodent chow and autoclaved water were available ad libitum. Animal procedures were conducted in strict accordance with the guidelines of the European Communities Council Directive 2010/63/E.U and approved by the Lithuania State Food and Veterinary Service, Animal Ethics Experimentation Committee (Nr. G2-237).

### 4.2. FMT Procedure

**Figure 5:**
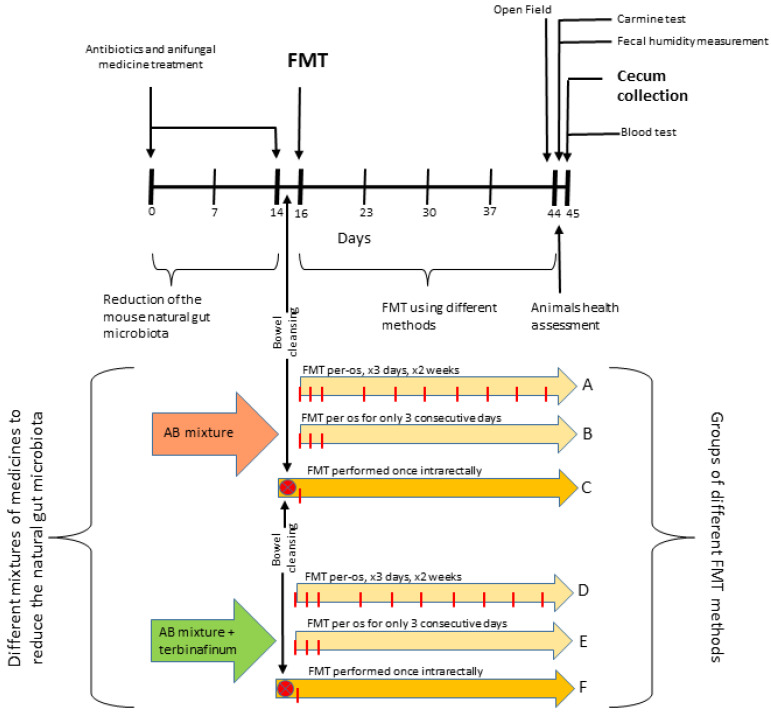
Schematic representation of the experiment timeline and structure.

A schematic representation of the experiment timeline and structure is presented in Figure 5.

The animals were divided into 6 groups (A, B, C, D, E, F) and exposed to different FMT techniques.

Gut-microbiota-reducing drugs were administered with drinking water 14 days before FMT as previously described [23]. Briefly, animals in groups A, B, and C received an antibiotic cocktail (AB): ampicillin and metronidazole (both 1 g/L), vancomycin (500 mg/L), ciprofloxacin HCl (200 mg/L), and imipenem (250 mg/L); animals in groups D, E, and F received the same antibiotic mixture plus an additional antifungal drug, terbinafine (200 mg/L) (AB + T).

A human stool sample was obtained from a 7-year-old healthy male individual. Permission for experiments with human gut microbiota samples was given by the Lithuanian Bioethics Committee (Lithuanian Bioethical Committee approval no. 2022/4-1400-893). A fecal microbiota sample was prepared by transferring 1.1–1.3 g of fecal material into a 15 mL tube. Then, 10 mL of PBS/15% glycerol (or saline/15% glycerol) solution was added for every 1 g of fecal material. Afterwards, the solution was mechanically mixed on max power for approximately 5 min until it became homogenous. After a 5 min step in the centrifuge at 2000 rpm, the solution supernatant was stored at −80 °C until needed.

Animals in groups A, B, D, and E were exposed to FMT via oral gavage (per os—PO). Specifically, a sterile, 3 cm long gastric tube was attached to a 1 mL syringe with 200 µL of a prepared gut microbiota sample previously collected. The tube was introduced into the stomach through the mouth and esophagus, and the microbiota sample in the syringe was administered. Two different dosing intervals were used: for 3 consecutive days once per day (group B and group E); for 3 consecutive days once a day and then the same procedure 2 times a week until the end of the study (group A and group D).

Animals in groups C and F were exposed to FMT via enema (intrarectal—IR). Specifically, mice were anesthetized with isoflurane for 1 min, and then the gut microbiota was transferred. A metal sterile tube was placed on a syringe, and a sample of the gut microbiota was taken at 400 µL. The tube was inserted 0.5–1 cm into the rectum, and the sample was gently and slowly passed into the intestine (reaching the cecum) using a syringe. The mice were fasted for 24 h before the procedure. Intestinal cleansing (using “Fortrans solution”) was performed 6 and 12 h after the start of fasting.

### 4.3. Assessment of Behavioral and Physiological Parameters

#### 4.3.1. Open-Field Test

Anxiety-like behavior was analyzed by performing an open-field test, as previously described [24].

Prior to the open-field test, the animals were habituated to the test environment by keeping them in a cage in a similar room for 1 h. The test was performed by placing the animal in an empty, white uncovered box (40 × 40 × 30 cm) and using a strong light (500 lx) as a stress factor. 

Animals were recorded during the test for 10 min, and the following parameters were evaluated:

(i) Total track length: the distance the animal walked in the whole arena during the entire test (cm);
(ii) Activity (%) during the experiment: the time during which the animal was active in the arena, expressed as a percentage of the total time of the test;
(iii) Duration in center zone: the time spent in the center of the arena (small zone);
(iv) Velocity in center zone: speed of the animal in the center zone (cm/s);
(v) Track length in center zone: the distance the animal walked in the center zone during the whole test (cm);
(vi) Visits: the number of visits to the center zone, or the number of times the animal visited the center zone during the experiment.

#### 4.3.2. Whole Intestinal Transit

Whole intestinal transit was assessed as previously described [25]. Animals were administered carmine dye by oral gavage, and the latency for the excretion of the first red-colored fecal pellet was recorded.

#### 4.3.3. Pellet Humidity

The fecal water content was calculated as the difference in weight after desiccation of fecal pellets, as previously described [25].

#### 4.3.4. Blood Sampling and Analysis

Hematological parameters were assessed using an automatic veterinary hematology analyzer (Exigo EOS, Jainam Biomedical, Sweden), as previously described [26]. Briefly, 10 µL of blood was collected from the tip of the mouse tail using a microcapillary, and hematological analysis was performed. Hematological parameters like white blood cells (WBCs) (×10^9^/L), lymphocytes (LYMs) (×10^9^/L), monocytes (MONs) (×10^9^/L), granulocytes (GRANs) (×10^9^/L), red blood cells (RBCs) (×10^9^/L), hematocrit (HCT) (g/dL), and hemoglobin (HGB) (g/dL) were evaluated.

### 4.4. 16S and 18S rRNA Amplicon Sequencing and Data Processing

Starting with stool sample processing until the final sequencing data were obtained, the following steps were carried out: (i) extraction of genome DNA; (ii) PCR amplification; (iii) PCR product quantification and mixing; (iv) PCR product purification; (v) library preparation; and (vi) sequencing. In order to reduce bias and maintain the accuracy and reliability of the sequencing data, quality control was performed at each step of the procedure. 

PCR amplification of targeted regions was performed by using specific primers connected with barcodes. The PCR products with proper sizes were selected via 2% agarose gel electrophoresis. The same amount of PCR products from each sample was pooled, end-repaired, A-tailed, and further ligated with Illumina adapters.

The libraries were checked with Qubit and real-time PCR for quantification, and a bioanalyzer for size distribution detection. According to the effective library concentration and data amount required, quantified libraries were pooled and sequenced on an Illumina paired-end platform to generate 250 bp paired-end raw reads. 

Paired-end reads were assigned to samples based on their unique barcodes and truncated by cutting off the barcode and primer sequences. Paired-end reads were merged using FLASH (V1.2.7) [27] in order to obtain raw tags. Quality filtering of the raw tags was performed under specific filtering conditions to obtain high-quality clean tags [28] according to the Qiime [29] (V1.7.0) quality-controlled process.

The tags were compared with the reference database SILVA [30] version 138 using the UCHIME algorithm [31] to detect and remove chimera sequences [32]; then, the effective tags were finally obtained.

Sequence analysis was performed using Uparse software [33] (v7.0.1090), using all the effective tags. Sequences with ≥97% similarity were assigned to the same OTUs. The representative sequence for each OTU was screened for further annotation.

For each representative sequence, taxonomic assignment was performed with Qiime using the Mothur method against the SSU rRNA SILVA 138 database [34] for species annotation of each taxonomic rank (threshold: 0.8~1) [30] (kingdom, phylum, class, order, family, genus, species). For the 18S sequencing data, taxonomic assignment was conducted with the RDP method using Qiime and the Silva database.

To obtain the phylogenetic relationship of all OTU representative sequences, the software MUSCLE [35] (Version 3.8.31) was used to compare multiple sequences rapidly.

OTU abundance information was normalized using a standard of sequence number corresponding to the sample with the least sequences. 

### 4.5. Gut Microbiota Analysis

The analysis of the 16S and 18S data was conducted with R (version 4.2.3) and R Studio (version 2023.03.0). For the analysis of the 18S data, only the fungal portion of the gut microbiota was considered.

Alpha diversity was assessed by calculating the Chao1 [36], Shannon [37], and Simpson [38] indices over raw feature counts, and differences between groups were tested with linear regression models, with *p* < 0.05 deemed significant.

Beta diversity was tested by calculating the Euclidean distance over centered log ratio (CLR)-transformed counts (Aitchison distance) [39] at the genus level, including only features with a total relative abundance ≥ 0.001 in at least 10% of the observations. Differences in fecal microbiota community dissimilarity between groups were assessed with permutational multivariate analysis of variance (PERMANOVA) on the computed Aitchison distance using the “adonis2” function of the vegan package (version 2.6-2) (https://CRAN.R-project.org/package=vegan, accessed on 27 June 2023).

The differences in the gut microbiota composition at the phylum and genus levels across the different groups were assessed through general linear models (GLMs) implemented through the package MaAsLin2 (version 1.10.0) [40] over CLR-transformed counts at the genus level, considering only features with a total relative abundance ≥ 0.001 in at least 10% of the observations. Only differential abundant features with Benjamini–Hochberg-adjusted *p*-values < 0.05 were considered significant.

**Figure 6:**
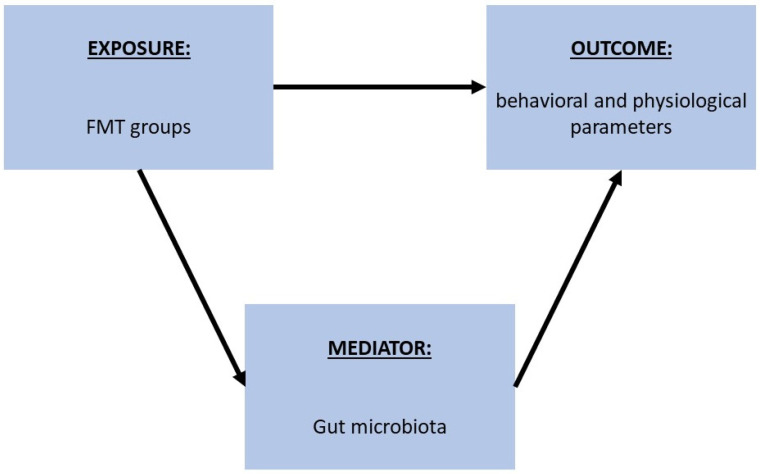
Schematic representation of the process followed to analyze the potential mediation of gut microbiota in the association between FMT techniques and different behavioral and physiological parameters. First, the association between FMT groups and different behavioral and physiological parameters was tested through a linear regression, and then the association between the parameters of interest and gut microbiota features that showed an association across the same FMT groups was tested.

For the current study, gut microbiota analysis was conducted as follows: (i) testing differences in alpha diversity, and differences in main phylum and genus abundances between the human donor (G) and FMT groups (A, B, C, D, E, F); (ii) testing differences in alpha and beta diversity between the different FMT groups, gut decontamination treatments (AB, AB + T), administrations (PO, IR), and dosing intervals (3 × 2 week time/day, 3 time/day, 1 time/day); (iii) testing the association between the different FMT groups and different behavioral and physiological parameters, and the possible mediation of the gut microbiota in these potential interactions (Figure 6). 

Specifically, we first tested the association between the FMT groups and the different behavioral and physiological parameters through linear regression models. Then, according to the results, we tested the association between the variables of interest and gut microbiota features that showed an association across the same groups (from the MaAsLin2 results), in order to evaluate the possible mediation of these features in the association between the FMT groups and behavioral and physiological parameters.

## Figures and Tables

**Table 1:** Characteristics and behavioral and physiological parameters of the animals included in this study, and within-group differences.

	Group A	Group B	Group C	Group D	Group E	Group F
Gut decontamination treatment	AB	AB	AB	AB + T	AB + T	AB + T
Drug administration	PO	PO	IR	PO	PO	IR
Dosing interval (times/day)	1 × 3 days × 2 weeks	1 × 3 days	1	1 × 3 days × 2 weeks	1 × 3 days	1
Intestinal transit time (m)	230.0 ± 41.0	197.7 ± 41.9 ^C^	261.2 ± 54.6 ^B,D^	195.6 ± 24.2 ^C^	209.2 ± 39.5	215.3 ± 56.7
Pellet humidity (water %)	64.0 ± 7.7	63.6 ± 8.7	66.8 ± 11.0	64.6 ± 8.2	55.7 ± 14.5	63.3 ± 11.3
WBCs (×10^9^/L)	18.8 ± 3.1	17.3 ± 3.2	17.1 ± 2.7	18.9 ± 4.3	19.2 ± 4.0	20.6 ± 2.3
LYMs (×10^9^/L)	14.6 ± 2.2	13.7 ± 2.3	13.5 ± 2.5	15.1 ± 3.5	15.5 ± 3.1	16.5 ± 1.7
MONs (×10^9^/L)	0.9 ± 0.2	0.9 ± 0.2	0.9 ± 0.2	0.9 ± 0.3	1.0 ± 0.3	1.0 ± 0.2
GRANs (×10^9^/L)	3.2 ± 0.9	2.7 ± 0.7	2.7 ± 0.5	2.8 ± 0.8	2.8 ± 0.8	3.0 ± 0.5
HGB (g/dL)	13.7 ± 0.8	14.1 ± 0.6	14.2 ± 0.5	13.7 ± 0.4	14.3 ± 1.0	14.2 ± 0.4
HCT (g/dL)	31.8 ± 1.8	32.3 ± 1.5	32.4 ± 1.4	31.0 ± 0.9	32.5 ± 2.1	31.9 ± 1.0
PLTs (×10^9^/L)	520.3 ± 179.9	689.7 ± 216.5	623.0 ± 154.1	600.4 ± 226.8	491.2 ± 124.0	604.0 ± 128.9
RBCs (×10^9^/L)	8.9 ± 0.5	9.0 ± 0.3	9.0 ± 0.4	8.6 ± 0.2	9.1 ± 0.6	8.9 ± 0.3
Total track length (cm)	3973.4 ± 407.0 ^B,E^	4623.3 ± 565.2 ^A^	4299.1 ± 380.8 ^E^	4104.4 ± 323.6 ^E^	4996.4 ± 588.9 ^A,C,D^	4483.8 ± 598.7
Activity during the experiment (%)	55.8 ± 4.3 ^E^	60.3 ± 4.0	57.8 ± 2.9	57.1 ± 2.9	60.9 ± 3.9 ^A^	58.9 ± 5.1
Duration (s)	104.0 ± 35.6	99.6 ± 18.2	105.2 ± 28.3	93.5 ± 18.5	80.9 ± 28.3	84.8 ± 24.9
Velocity (cm/s)	9.2 ± 1.8 ^E^	10.6 ± 1.9	9.3 ± 1.4 ^E^	9.8 ± 1.5	12.1 ± 2.4 ^A,C^	10.6 ± 2.0
Track length (cm)	918.3 ± 250.1	1036.5 ± 188.8	949.8 ± 185.9	908.6 ± 179.6	932.7 ± 205.2	870.1 ± 206.2
Visits to the center	56.2 ± 18.7	57.8 ± 9.4	49.5 ± 7.9	50.2 ± 9.3	54.6 ± 9.8	47.8 ± 11.4

AB, antibiotic cocktail; AB + T, antibiotic cocktail plus terbinafine; PO, per os; IR, intrarectal; WBCs, white blood cells; LYMs, lymphocytes; MONs, monocytes; GRANs, granulocytes; HGB, hemoglobin; HCT, hematocrit; PLTs, platelets; RBCs, red blood cells. Within-group differences were tested through a linear regression. ^A^ *p* < 0.05 vs. group A, ^B^ *p* < 0.05 vs. group B, ^C^ *p* < 0.05 vs. group C, ^D^ *p* < 0.05 vs. group D, ^E^ *p* < 0.05 vs. group E.

**Table 2:** Differences between the human donor and FMT groups in calculated alpha diversity indices Chao1, Shannon, and Simpson, in the 16S dataset. Differences were tested through a linear regression, and *p* < 0.05 is indicated in bold.

Index	FMT Group vs. Donor	Coef.	Std. Error	t Value	Pr (>|t|)
Chao1	A	−23.489	15.183	−1.547	0.1331
Chao1	B	−32.258	15.183	−2.125	**0.0426**
Chao1	C	−21.345	15.183	−1.406	0.1708
Chao1	D	−28.109	15.398	−1.826	0.0786
Chao1	E	−19.778	15.398	−1.284	0.2095
Chao1	F	−11.999	15.183	−0.790	0.4360
Shannon	A	−0.788	0.194	−4.053	**0.0004**
Shannon	B	−0.868	0.194	−4.462	**0.0001**
Shannon	C	−0.785	0.194	−4.036	**0.0004**
Shannon	D	−0.825	0.197	−4.181	**0.0003**
Shannon	E	−0.819	0.197	−4.155	**0.0003**
Shannon	F	−0.777	0.194	−3.996	**0.0004**
Simpson	A	−0.093	0.032	−2.874	**0.0077**
Simpson	B	−0.098	0.032	−3.034	**0.0052**
Simpson	C	−0.105	0.032	−3.240	**0.0031**
Simpson	D	−0.088	0.033	−2.674	**0.0124**
Simpson	E	−0.104	0.033	−3.174	**0.0036**
Simpson	F	−0.087	0.032	−2.703	**0.0115**

**Table 3:** Significant differences across the FMT groups in calculated alpha diversity indices Chao1, Shannon, and Simpson, in the 16S dataset. Differences were tested through a linear regression, and *p* < 0.05 was deemed significant.

Index	FMT Group	vs. Group	Coef.	Std. Error	t Value	Pr (>|t|)
Shannon	A	F	−0.396	0.163	2.431	0.022
Shannon	C	F	−0.410	0.163	2.518	0.018
Shannon	E	F	−0.433	0.171	2.538	0.017
Simpson	A	F	−0.030	0.014	−2.109	0.044
Simpson	B	F	−0.031	0.014	−2.144	0.041
Simpson	E	F	−0.033	0.015	−2.185	0.037

**Table 4:** Differences in the gut fungal community at the genus level between the human donor and FMT groups.

Fungi Genera	FMT Group vs. Donor	Coef.	Std. Error	pval	FDR
Nakaseomyces/Candida clade	A	−5.2455	1.1269	7.121 × 10^−^³	0.0273
Nakaseomyces/Candida clade	C	−4.6934	1.1269	0.0003	0.0308
Saccharomyces	A	−7.0721	1.7246	0.0003	0.0308
Funneliformis	A	−5.0104	1.1637	0.0002	0.0308
Nakaseomyces/Candida clade	B	−4.2976	1.1269	0.0007	0.0379
Tremellales	A	−3.7649	0.9642	0.0005	0.0379
Funneliformis	C	−4.4397	1.1637	0.0007	0.0379

The results of the generalized linear model are shown. Reference: G (human donor). FDR < 0.05 was deemed significant.

**Table 5:** Significant differences across the different FMT groups in calculated alpha diversity indices Chao1, Shannon, and Simpson, in the 18S dataset. Differences were tested through a linear regression, with *p* < 0.05.

Index	FMT Group	vs. Group	Coef.	Std. Error	t Value	Pr (>|t|)
Chao1	B	F	−467.672	177.976	2.628	0.014
Shannon	B	E	−1.688	0.647	−2.609	0.014
Shannon	B	F	−2.266	0.617	3.674	0.001
Shannon	C	E	−1.581	0.647	−2.444	0.021
Shannon	C	F	−2.159	0.617	3.500	0.002
Shannon	D	F	−1.365	0.647	2.110	0.044
Simpson	B	E	−0.336	0.153	−2.192	0.037
Simpson	B	F	−0.419	0.146	2.863	0.008
Simpson	C	E	−0.347	0.153	−2.259	0.032
Simpson	C	F	−0.429	0.146	2.933	0.007

**Table 6:** Differences in the Aitchison distance calculated in the 18S dataset across the different FMT conditions. Differences were assessed through a PERMANOVA test, and *p* < 0.05 was deemed significant and indicated in bold.

FMT Condition	Df	Sum of sqs	R2	F	Pr (>F)
Gut decontamination treatment	1	1111.382	0.072	2.632	**0.002**
Drug administration	1	443.481	0.029	1.050	0.382
Dosing interval	1	372.913	0.024	0.883	0.611
FMT group	2	1642.543	0.107	1.945	**0.002**
Residual	28	11,822.200	0.768		
Total	33	15,392.520	1.000		

**Table 7:** Differences in gut fungal genera between the FMT groups.

Fungi Genera	FMT Group	vs. Group	Coef.	Std. Error	pval	FDR
*Symmetrospora*	C	A	3.0442	0.6683	0.0001	0.0100
*Melanopsichium*	C	A	−2.3897	0.6184	0.0006	0.0299
*Cheilymenia*	D	A	3.1651	0.6334	0.0001	0.0090
*Melanopsichium*	D	A	−2.8680	0.6486	0.0001	0.0107
*Nakaseomyces/Candida clade*	D	A	2.2211	0.6281	0.0014	0.0459
*Curvularia*	D	A	1.8679	0.5365	0.0017	0.0481
*Tremellales*	E	A	2.5559	0.5432	0.0001	0.0099
*Camptobasidiaceae*	E	A	3.0886	0.7396	0.0003	0.0167
*Paramicrosporidium*	E	A	−3.2808	0.9034	0.0011	0.0447
*Tranzscheliella*	E	A	2.5525	0.7144	0.0013	0.0459
*Kondoa*	F	A	2.1965	0.5729	0.0007	0.0299
*Symmetrospora*	C	B	3.0315	0.6683	0.0001	0.0315
*Symmetrospora*	F	C	−2.9404	0.6683	0.0001	0.0152
*Camptobasidiaceae*	E	C	2.9426	0.7396	0.0004	0.0356
*Cheilymenia*	E	D	−2.7530	0.6616	0.0003	0.0290

The results of the generalized linear model are shown. FDR < 0.05 was deemed significant.

**Table 8:** Differences in gut fungal genera between animals exposed to the different gut decontamination treatments.

Fungi Genera	Treatment	Coef.	Std. Error	pval	FDR
*Doratomyces*	AB + T	−1.9285	0.5875	0.0025	0.0285
*Hymenochaetaceae*	AB + T	−1.7169	0.5449	0.0035	0.0285
*Paramicrosporidium*	AB + T	−1.6149	0.5296	0.0046	0.0293
*LKM11*	AB + T	−1.5834	0.5775	0.0099	0.0488
*Saccharomycopsis*	AB + T	−1.5729	0.5072	0.0040	0.0285
*Tremellales*	AB + T	1.1677	0.3409	0.0017	0.0285
*Funneliformis*	AB + T	1.2020	0.3648	0.0024	0.0285
*Tranzscheliella*	AB + T	1.3443	0.4297	0.0037	0.0285
*Nakaseomyces.Candida_clade*	AB + T	1.4258	0.3524	0.0003	0.0197
*Camptobasidiaceae*	AB + T	1.4964	0.4598	0.0027	0.0285
*Saccharomyces*	AB + T	1.5507	0.5733	0.0109	0.0496
*Suillus*	AB + T	1.8622	0.6362	0.0063	0.0364
*Neurospora*	AB + T	1.8665	0.5694	0.0025	0.0285
*Ogataea.Candida_clade*	AB + T	2.0946	0.7261	0.0070	0.0371

AB + T, antibiotic cocktail + terbinafine treatment. The results of the generalized linear model are shown. FDR < 0.05 was deemed significant.

**Table 9:** Association between the selected fungal genera and behavioral parameters of interest.

Fungi Genera	Behavioral Parameter	Association	Coef.	Std. Error	t Value	Pr (>|t|)
*Tremellales*	Total track length	E vs. A	0.0009	0.0003	2.9330	**0.0062**
*Camptobasidiaceae*	Total track length	E vs. A,C	0.0013	0.0004	3.1740	**0.0033**
*Paramicrosporidium*	Total track length	E vs. A	−0.0004	0.0005	−0.7620	0.4520
*Tranzscheliella*	Total track length	E vs. A	0.0009	0.0004	2.2230	**0.0334**
*Cheilymenia*	Total track length	E vs. D	−0.0002	0.0004	−0.5470	0.5880
*Tremellales*	Activity during the exp.	E vs. A	0.0808	0.0476	1.6960	0.0997
*Camptobasidiaceae*	Activity during the exp.	E vs. A	0.1008	0.0637	1.5840	0.1230
*Paramicrosporidium*	Activity during the exp.	E vs. A	−0.0418	0.0746	−0.5600	0.5790
*Tranzscheliella*	Activity during the exp.	E vs. A	0.0724	0.0596	1.2160	0.2330
*Tremellales*	Velocity	E vs. A	0.1908	0.0970	1.9660	0.0580
*Camptobasidiaceae*	Velocity	E vs. A,C	0.4199	0.1146	3.6630	**0.0009**
*Paramicrosporidium*	Velocity	E vs. A	0.0955	0.1540	0.6200	0.5390
*Tranzscheliella*	Velocity	E vs. A	0.1027	0.1245	0.8250	0.4154
*Cheilymenia*	Velocity	E vs. D	−0.2727	0.1582	−1.7250	0.0943

The results of the linear regression are shown. *p* < 0.05 was deemed significant and indicated in bold.

## Data Availability

The data presented in this study are openly available on FigShare at https://doi.org/10.6084/m9.figshare.23600787.v2, accessed on 29 June 2023.

## Supplementary Materials

The following supporting information can be downloaded at: https://www.mdpi.com/article/10.3390/ijms241512005/s1.
