# Supplementary material for: A Comparison of Methods of Gut Microbiota Transplantation for Preclinical Studies
Source: Int J Mol Sci. 2023 Jul 26;24(15):12005. doi: 10.3390/ijms241512005 (PMC10418867; doi:10.3390/ijms241512005)
Supplement: Supplementary file 1 [file ijms-24-12005-s001.zip › ijms-2500330-supplementary.pdf]

Comparison of the methods for the gut microbiota transplantation for preclinical studies

Jonas Mingaila [1,#], Alessandro Atzeni [1,#,*], Aurelijus Burokas [1,*]

[1] Department of Biological Models, Institute of Biochemistry, Life Sciences Center, Vilnius University, Sauletekio Ave. 7, LT-10257 Vilnius, Lithuania

[#] Equally contributed
* Correspondence: alessandro.atzeni@gmc.vu.lt, aureliju.burokas@gmc.vu.lt

Supplemental material

Table S1. Significant differential abundant gut bacterial phyla between human donor and different FMT groups. Results of generalized linear model. FDR < 0.05 deemed as significant.

Bacteria phyla	FMT group vs donor	Coef.	Std. error	pval	FDR
Euryarchaeota	B	-6.5400	0.9555	1.9496E-07	4.2373E-06
Euryarchaeota	C	-6.5724	0.9555	1.7847E-07	4.2373E-06
Euryarchaeota	F	-6.4281	0.9555	2.6483E-07	4.2373E-06
Euryarchaeota	A	-6.0447	0.9555	7.6475E-07	7.3416E-06
Euryarchaeota	E	-6.2105	0.9691	6.1407E-07	7.3416E-06
Euryarchaeota	D	-6.0132	0.9691	1.0562E-06	8.4493E-06
Bacteroidota	E	2.4414	0.5772	0.0002	0.0016
Bacteroidota	F	2.0804	0.5691	0.0010	0.0063
Bacteroidota	A	1.8234	0.5691	0.0034	0.0147
Bacteroidota	B	1.8317	0.5691	0.0032	0.0147
Bacteroidota	C	1.8356	0.5691	0.0032	0.0147
Actinobacteriota	B	-3.6735	1.1727	0.0040	0.0161
Actinobacteriota	D	-3.4666	1.1893	0.0069	0.0256
Bacteroidota	D	1.6451	0.5772	0.0081	0.0278

Table S2. Significant differential abundant gut bacterial genera between human donor and FMT groups. Results of generalized linear model. FDR< 0.05 deemed as significant

Bacteria genera	FMT group vs donor	Coef.	Std. Error	pval	FDR
Methanobrevibacter	A	-7.3772	0.9407	1.527E-08	1.9698E-06
Eubacterium_brachy_group	A	2.8232	0.6064	7.1035E-05	0.0026
Dorea	A	-3.6431	0.8167	0.0001	0.0034
Prevotella	A	-4.4534	1.0055	0.0001	0.0034
Candidatus_Arthromitus	A	5.5625	1.3617	0.0003	0.0055
Lachnospiraceae_NK4A136_group	A	2.6760	0.6685	0.0004	0.0057
Bacteroides	A	2.8482	0.7058	0.0004	0.0057
Oscillibacter	A	2.8473	0.7192	0.0005	0.0062
Senegalimassilia	A	-4.2778	1.0896	0.0005	0.0066
Erysipelotrichaceae_UCG.003	A	-5.8194	1.4868	0.0005	0.0067
GCA.900066575	A	2.5647	0.6652	0.0006	0.0071
Collinsella	A	-5.5075	1.4906	0.0009	0.0077
Ruminococcaceae	A	2.8307	0.7501	0.0008	0.0077
Parabacteroides	A	2.4367	0.6601	0.0010	0.0077
Colidextribacter	A	2.3140	0.6610	0.0016	0.0099
Alistipes	A	2.6119	0.7441	0.0015	0.0099
Lachnospiraceae_UCG.001	A	3.0665	0.8829	0.0017	0.0103
Christensenellaceae_R.7_group	A	-3.4046	1.0431	0.0029	0.0122
Ruminococcus_gauvreauii_group	A	-5.5150	1.6976	0.0030	0.0124
Acetatifactor	A	5.2191	1.6162	0.0032	0.0126
Lachnospiraceae_UCG.006	A	2.3297	0.7253	0.0033	0.0127
Clostridia_vadinBB60_group	A	2.2578	0.7117	0.0036	0.0137
Coprococcus	A	-5.3367	1.7008	0.0040	0.0144
Lachnoclostridium	A	2.5405	0.8209	0.0044	0.0155
Faecalibacterium	A	-5.1626	1.6772	0.0046	0.0159
Fusicatenibacter	A	-6.7834	2.2177	0.0049	0.0162
Enterorhabdus	A	2.1891	0.7158	0.0049	0.0162
Intestinibacter	A	-6.9125	2.2568	0.0048	0.0162
Butyricimonas	A	2.9130	0.9627	0.0053	0.0172
Eubacterium_xylanophilum_group	A	2.1737	0.7196	0.0053	0.0173
Subdoligranulum	A	-5.7691	1.9737	0.0068	0.0204
Ruminococcus_torques_group	A	-5.3180	1.8240	0.0069	0.0204
Incertae_Sedis	A	1.6078	0.5519	0.0070	0.0204
Sarcina	A	-6.4455	2.2656	0.0082	0.0233
A2	A	3.2878	1.1678	0.0088	0.0243
UCG.003	A	2.5370	0.9073	0.0092	0.0243
Monoglobus	A	1.9247	0.6979	0.0101	0.0255
Romboutsia	A	-4.6393	1.8130	0.0162	0.0366
Butyricicoccus	A	1.7646	0.6895	0.0162	0.0366
Odoribacter	A	2.1696	0.8511	0.0166	0.0372

Eubacterium_hallii_group	A	-4.6522	1.8303	0.0168	0.0376
Adlercreutzia	A	-2.5454	1.0247	0.0192	0.0410
Eubacterium_siraeum_group	A	2.6174	1.0516	0.0190	0.0410
Methanobrevibacter	B	-7.6966	0.9407	6.6044E-09	1.5933E-06
Dorea	B	-3.8571	0.8167	5.9131E-05	0.0024
Eubacterium_brachy_group	B	2.9141	0.6064	4.7164E-05	0.0024
Prevotella	B	-4.7347	1.0055	6.1516E-05	0.0024
Lachnospiraceae_NK4A136_group	B	3.0105	0.6685	0.0001	0.0034
Bacteroides	B	3.1371	0.7058	0.0001	0.0034
GCA.900066575	B	2.9055	0.6652	0.0002	0.0038
Candidatus_Arthromitus	B	5.8758	1.3617	0.0002	0.0040
Oscillibacter	B	3.0911	0.7192	0.0002	0.0040
Erysipelotrichaceae_UCG.003	B	-6.2871	1.4868	0.0002	0.0043
Collinsella	B	-6.1555	1.4906	0.0003	0.0051
Senegalimassilia	B	-4.3900	1.0896	0.0004	0.0057
Lachnoclostridium	B	3.1593	0.8209	0.0006	0.0071
Parabacteroides	B	2.5492	0.6601	0.0006	0.0071
Alistipes	B	2.8789	0.7441	0.0006	0.0071
Acetatifactor	B	5.9867	1.6162	0.0009	0.0077
Lachnospiraceae_UCG.001	B	3.2337	0.8829	0.0010	0.0081
Ruminococcus_gauvreauii_group	B	-6.1510	1.6976	0.0011	0.0085
Colidextribacter	B	2.3931	0.6610	0.0012	0.0085
Christensenellaceae_R.7_group	B	-3.7645	1.0431	0.0012	0.0086
Ruminococcaceae	B	2.6983	0.7501	0.0012	0.0088
Intestinibacter	B	-7.9029	2.2568	0.0016	0.0099
Ruminococcus_torques_group	B	-6.2779	1.8240	0.0018	0.0103
Fusicatenibacter	B	-7.5996	2.2177	0.0019	0.0104
Adlercreutzia	B	-3.5069	1.0247	0.0019	0.0104
Faecalibacterium	B	-5.7387	1.6772	0.0019	0.0104
Clostridia_vadinBB60_group	B	2.4313	0.7117	0.0020	0.0104
Odoribacter	B	2.8963	0.8511	0.0020	0.0107
Enterorhabdus	B	2.4256	0.7158	0.0021	0.0107
Butyricimonas	B	3.2306	0.9627	0.0023	0.0112
Incertae_Sedis	B	1.8454	0.5519	0.0024	0.0113
Sarcina	B	-7.4591	2.2656	0.0027	0.0120
UCG.010	B	3.1735	0.9648	0.0027	0.0120
Subdoligranulum	B	-6.4618	1.9737	0.0028	0.0121
A2	B	3.7693	1.1678	0.0032	0.0126
Eubacterium_xylanophilum_group	B	2.3256	0.7196	0.0031	0.0126
Coprococcus	B	-5.4836	1.7008	0.0032	0.0126
Lachnospiraceae_UCG.006	B	2.2987	0.7253	0.0037	0.0137
Butyricicoccus	B	2.1123	0.6895	0.0048	0.0162
Clostridium_sensu_stricto_1	B	-6.8374	2.2378	0.0049	0.0162

Anaerostipes	B	-4.8572	1.6173	0.0056	0.0175
Lactobacillus	B	3.7454	1.3030	0.0076	0.0220
UCG.003	B	2.5566	0.9073	0.0088	0.0243
Eubacterium_hallii_group	B	-5.1079	1.8303	0.0094	0.0243
Monoglobus	B	1.8838	0.6979	0.0117	0.0282
Anaerotruncus	B	1.9276	0.7221	0.0125	0.0298
Romboutsia	B	-4.5947	1.8130	0.0171	0.0381
ASF356	B	2.0723	0.8258	0.0182	0.0399
Methanobrevibacter	C	-7.5670	0.9407	9.2635E-09	1.5933E-06
Dorea	C	-3.8797	0.8167	5.483E-05	0.0024
Prevotella	C	-4.5481	1.0055	0.0001	0.0034
Eubacterium_brachy_group	C	2.6123	0.6064	0.0002	0.0040
Erysipelotrichaceae_UCG.003	C	-5.9783	1.4868	0.0004	0.0057
Bacteroides	C	2.8292	0.7058	0.0004	0.0057
Oscillibacter	C	2.7867	0.7192	0.0006	0.0071
Senegalimassilia	C	-4.1736	1.0896	0.0007	0.0071
Candidatus_Arthromitus	C	5.0373	1.3617	0.0009	0.0077
Ruminococcaceae	C	2.7876	0.7501	0.0009	0.0077
Collinsella	C	-5.3478	1.4906	0.0013	0.0089
Christensenellaceae_R.7_group	C	-3.6320	1.0431	0.0017	0.0102
Parabacteroides	C	2.2975	0.6601	0.0017	0.0102
Lachnoclostridium	C	2.7900	0.8209	0.0020	0.0107
Clostridia_vadinBB60_group	C	2.4139	0.7117	0.0021	0.0107
Ruminococcus_gauvreauii_group	C	-5.7277	1.6976	0.0022	0.0109
GCA.900066575	C	2.2290	0.6652	0.0023	0.0112
Alistipes	C	2.4856	0.7441	0.0024	0.0113
Acetatifactor	C	5.3709	1.6162	0.0025	0.0116
Lachnospiraceae_NK4A136_group	C	2.2035	0.6685	0.0027	0.0120
Incertae_Sedis	C	1.7761	0.5519	0.0033	0.0127
Faecalibacterium	C	-5.3183	1.6772	0.0037	0.0137
Ruminococcus_torques_group	C	-5.7529	1.8240	0.0038	0.0140
Intestinibacter	C	-7.0876	2.2568	0.0040	0.0144
Colidextribacter	C	1.9917	0.6610	0.0054	0.0173
Subdoligranulum	C	-5.8920	1.9737	0.0058	0.0180
Fusicatenibacter	C	-6.5432	2.2177	0.0063	0.0193
Lachnospiraceae_UCG.006	C	2.1163	0.7253	0.0069	0.0204
Sarcina	C	-6.5028	2.2656	0.0077	0.0221
Butyricimonas	C	2.7074	0.9627	0.0089	0.0243
Lachnospiraceae_UCG.001	C	2.4710	0.8829	0.0092	0.0243
A2	C	3.2737	1.1678	0.0091	0.0243
Eubacterium_xylanophilum_group	C	2.0080	0.7196	0.0094	0.0243
UCG.010	C	2.6791	0.9648	0.0097	0.0250
Coprococcus	C	-4.7050	1.7008	0.0099	0.0255

Adlercreutzia	C	-2.8298	1.0247	0.0100	0.0255
Barnesiella	C	2.1994	0.7978	0.0102	0.0255
Monoglobus	C	1.9177	0.6979	0.0104	0.0259
Odoribacter	C	2.2994	0.8511	0.0116	0.0282
Eubacterium_hallii_group	C	-4.8186	1.8303	0.0136	0.0320
Sphingomonas	C	3.7783	1.4959	0.0175	0.0387
Enterorhabdus	C	1.7848	0.7158	0.0188	0.0408
Eubacterium_siraeum_group	C	2.5851	1.0516	0.0204	0.0430
Methanobrevibacter	D	-7.7894	0.9540	6.889E-09	1.5933E-06
Dorea	D	-3.9558	0.8282	5.1141E-05	0.0024
Eubacterium_brachy_group	D	2.9563	0.6150	4.6986E-05	0.0024
Prevotella	D	-4.8514	1.0198	5.3874E-05	0.0024
Senegalimassilia	D	-4.7237	1.1051	0.0002	0.0041
Erysipelotrichaceae_UCG.003	D	-6.4344	1.5079	0.0002	0.0041
Bacteroides	D	3.0086	0.7158	0.0002	0.0045
Lachnospiraceae_NK4A136_group	D	2.7635	0.6780	0.0003	0.0055
Collinsella	D	-5.7756	1.5117	0.0007	0.0071
Lachnoclostridium	D	3.1358	0.8325	0.0008	0.0077
Candidatus_Arthromitus	D	5.1440	1.3811	0.0009	0.0077
Christensenellaceae_R.7_group	D	-3.9148	1.0579	0.0009	0.0077
Ruminococcaceae	D	2.8364	0.7608	0.0009	0.0077
Alistipes	D	2.8116	0.7547	0.0009	0.0077
Parabacteroides	D	2.4607	0.6695	0.0010	0.0079
Ruminococcus_gauvreauii_group	D	-6.2761	1.7216	0.0011	0.0083
GCA.900066575	D	2.4412	0.6746	0.0012	0.0085
Lactobacillus	D	4.7343	1.3215	0.0013	0.0089
Oscillibacter	D	2.5850	0.7294	0.0014	0.0095
Intestinibacter	D	-8.0814	2.2889	0.0015	0.0096
A2	D	4.1024	1.1844	0.0017	0.0103
Fusicatenibacter	D	-7.7358	2.2492	0.0018	0.0103
Ruminococcus_torques_group	D	-6.3732	1.8499	0.0018	0.0103
Clostridia_vadinBB60_group	D	2.4927	0.7218	0.0018	0.0103
Faecalibacterium	D	-5.8596	1.7010	0.0018	0.0103
Lachnospiraceae_UCG.001	D	3.0243	0.8954	0.0022	0.0109
Odoribacter	D	2.8933	0.8632	0.0023	0.0112
Enterorhabdus	D	2.4042	0.7260	0.0026	0.0118
Sarcina	D	-7.6012	2.2977	0.0026	0.0118
Lachnospiraceae_UCG.006	D	2.4180	0.7356	0.0027	0.0120
Subdoligranulum	D	-6.5661	2.0017	0.0028	0.0121
Butyricimonas	D	3.2005	0.9764	0.0028	0.0121
Coprococcus	D	-5.6126	1.7249	0.0030	0.0124
Colidextribacter	D	2.1557	0.6703	0.0033	0.0127
Eubacterium_xylanophilum_group	D	2.3046	0.7298	0.0038	0.0140

Incertae_Sedis	D	1.7528	0.5597	0.0040	0.0144
Clostridium_sensu_stricto_1	D	-6.8845	2.2696	0.0052	0.0170
Acetatifactor	D	4.9034	1.6391	0.0057	0.0179
UCG.010	D	2.8522	0.9785	0.0069	0.0204
Anaerostipes	D	-4.5905	1.6403	0.0092	0.0243
Eubacterium_hallii_group	D	-5.2078	1.8562	0.0090	0.0243
Anaerotruncus	D	2.0017	0.7324	0.0107	0.0265
ASF356	D	2.2664	0.8375	0.0115	0.0281
UCG.003	D	2.3979	0.9202	0.0145	0.0337
Monoglobus	D	1.8292	0.7078	0.0153	0.0352
Butyricicoccus	D	1.7262	0.6993	0.0199	0.0422
Adlercreutzia	D	-2.4869	1.0392	0.0237	0.0486
Methanobrevibacter	E	-7.2661	0.9540	2.6899E-08	2.5756E-06
Prevotella	E	-4.5391	1.0198	0.0001	0.0034
Dorea	E	-3.4691	0.8282	0.0003	0.0045
Bacteroides	E	2.9015	0.7158	0.0004	0.0057
Lachnospiraceae_NK4A136_group	E	2.6035	0.6780	0.0006	0.0071
Erysipelotrichaceae_UCG.003	E	-5.6588	1.5079	0.0008	0.0077
Eubacterium_brachy_group	E	2.2794	0.6150	0.0009	0.0077
Senegalimassilia	E	-3.9121	1.1051	0.0014	0.0095
GCA.900066575	E	2.3634	0.6746	0.0016	0.0099
Lachnoclostridium	E	2.7708	0.8325	0.0025	0.0115
Clostridia_vadinBB60_group	E	2.3570	0.7218	0.0029	0.0122
Ruminococcaceae	E	2.4736	0.7608	0.0030	0.0124
Collinsella	E	-4.8887	1.5117	0.0031	0.0126
Parabacteroides	E	2.1291	0.6695	0.0036	0.0137
Ruminococcus_gauvreauii_group	E	-5.4538	1.7216	0.0037	0.0137
Oscillibacter	E	2.2851	0.7294	0.0040	0.0144
Christensenellaceae_R.7_group	E	-3.3093	1.0579	0.0041	0.0144
Alistipes	E	2.3401	0.7547	0.0044	0.0153
Intestinibacter	E	-6.8987	2.2889	0.0054	0.0173
Odoribacter	E	2.6003	0.8632	0.0054	0.0173
Eubacterium_siraeum_group	E	3.1836	1.0665	0.0058	0.0180
Ruminococcus_torques_group	E	-5.4877	1.8499	0.0061	0.0187
Butyricimonas	E	2.8907	0.9764	0.0062	0.0189
A2	E	3.3940	1.1844	0.0078	0.0223
Fusicatenibacter	E	-6.3915	2.2492	0.0083	0.0233
Lachnospiraceae_UCG.010	E	3.6054	1.2732	0.0085	0.0237
Eubacterium_xylanophilum_group	E	2.0509	0.7298	0.0089	0.0243
Faecalibacterium	E	-4.7812	1.7010	0.0089	0.0243
Candidatus_Arthromitus	E	3.8577	1.3811	0.0093	0.0243
Subdoligranulum	E	-5.6010	2.0017	0.0092	0.0243
Coprococcus	E	-4.7533	1.7249	0.0102	0.0255

Sarcina	E	-6.2143	2.2977	0.0115	0.0281
Lachnospiraceae_UCG.006	E	1.9636	0.7356	0.0125	0.0298
Colidextribacter	E	1.7742	0.6703	0.0132	0.0311
Lachnospiraceae_UCG.001	E	2.3168	0.8954	0.0152	0.0351
Monoglobus	E	1.8151	0.7078	0.0160	0.0365
Clostridium_sensu_stricto_1	E	-5.6934	2.2696	0.0182	0.0399
Acetatifactor	E	4.0942	1.6391	0.0186	0.0406
UCG.010	E	2.4310	0.9785	0.0192	0.0410
Anaerotruncus	E	1.8165	0.7324	0.0194	0.0412
ASF356	E	2.0450	0.8375	0.0212	0.0445
Eubacterium_hallii_group	E	-4.5158	1.8562	0.0216	0.0450
Ruminococcus	E	1.0768	0.4492	0.0234	0.0486
Blautia	E	-1.5955	0.6666	0.0236	0.0486
UCG.003	E	2.1905	0.9202	0.0243	0.0498
Methanobrevibacter	F	-7.1244	0.9407	2.9948E-08	2.5756E-06
Lachnospiraceae_NK4A136_group	F	2.4876	0.6685	0.0009	0.0077
Eubacterium_brachy_group	F	2.2516	0.6064	0.0009	0.0077
Dorea	F	-2.9031	0.8167	0.0014	0.0094
Prevotella	F	-3.4802	1.0055	0.0017	0.0103
Bacteroides	F	2.4237	0.7058	0.0019	0.0104
GCA.900066575	F	2.2301	0.6652	0.0023	0.0112
Collinsella	F	-4.8268	1.4906	0.0031	0.0126
Senegalimassilia	F	-3.3683	1.0896	0.0045	0.0155
Lachnospiraceae_UCG.006	F	2.2253	0.7253	0.0047	0.0162
Erysipelotrichaceae_UCG.003	F	-4.4775	1.4868	0.0055	0.0173
Ruminococcaceae	F	2.2040	0.7501	0.0065	0.0197
Alistipes	F	2.1660	0.7441	0.0070	0.0204
Lachnoclostridium	F	2.3649	0.8209	0.0075	0.0218
Eubacterium_xylanophilum_group	F	2.0429	0.7196	0.0083	0.0234
Parabacteroides	F	1.8241	0.6601	0.0100	0.0255
Oscillibacter	F	1.9662	0.7192	0.0107	0.0265
Christensenellaceae_R.7_group	F	-2.8051	1.0431	0.0119	0.0288
Butyricimonas	F	2.5689	0.9627	0.0125	0.0298
Clostridia_vadinBB60_group	F	1.8925	0.7117	0.0128	0.0303
Monoglobus	F	1.8342	0.6979	0.0138	0.0322
Colidextribacter	F	1.6997	0.6610	0.0157	0.0361
Candidatus_Arthromitus	F	3.4797	1.3617	0.0163	0.0368
Blautia	F	-1.6431	0.6572	0.0186	0.0406
Ruminococcus_gauvreauii_group	F	-4.2224	1.6976	0.0191	0.0410
Acetatifactor	F	3.9420	1.6162	0.0213	0.0445

Table S3. Results of linear regression to test differences in alpha diversity indices Chao1, Shannon, and Simpson across different gut decontamination treatment, administration, and dosing interval in 16S dataset. Pr(>|t|)<0.05 deemed as significant.

Index	FMT condition	Coef.	Std. error	t value	Pr(>|t|)
Chao	AB+T treatment	18.270	19.100	0.956	0.346
Shannon	AB+T treatment	-0.131	0.102	-1.280	0.210
Simpson	AB+T treatment	-0.011	0.009	-1.268	0.214
Chao	PO administration	4.575	20.213	0.226	0.822
Shannon	PO administration	0.164	0.106	1.549	0.131
Simpson	PO administration	0.016	0.009	1.803	0.081
Chao	1 vs 3 (time/day)	8.190	23.853	0.343	0.734
Chao	1 vs 3x2 wk (time/day) dose	0.960	23.853	0.040	0.968
Chao	3x2 wk vs 3 (time/day) dose	7.231	24.366	0.297	0.769
Shannon	1 vs 3 (time/day) dose	0.162	0.125	1.298	0.204
Shannon	1 vs 3x2 wk (time/day) dose	0.165	0.125	1.323	0.196
Shannon	3x2 wk vs 3 (time/day) dose	-0.003	0.128	-0.024	0.981
Simpson	1 vs 3 (time/day) dose	0.017	0.010	1.672	0.105
Simpson	1 vs 3x2 wk (time/day) dose	0.014	0.010	1.384	0.176
Simpson	3x2 wk vs 3 (time/day) dose	0.003	0.011	0.281	0.780

Table S4. Differences in gut bacterial community at phylum level between FMT groups. Results of generalized linear model. Reference: group F. FDR < 0.05 deemed as significant.

Bacteria phyla	FMT group	Vs group	Coef.	Std. error	pval	FDR
Actinobacteriota	B	F	-2.4134	0.6228	0.0006	0.0205
Actinobacteriota	D	F	-2.1481	0.6532	0.0027	0.0281
Proteobacteria	D	F	-1.4241	0.4419	0.0032	0.0281
Verrucomicrobiota	C	F	3.3026	0.9594	0.0018	0.0281

Table S5. Differences in Aitchison distance calculated in 16S dataset between different FMT conditions assessed with PERMANOVA test.

**FMT condition**	**Df**	**Sum of sqs**	**R2**	**F**	**Pr(>F)**
Dosing interval	1	56.163	0.010	0.347	0.990
Gut decontamination treatment	1	249.941	0.044	1.546	0.141
Gut decontamination administration	1	303.558	0.054	1.878	0.091
FMT group	2	514.306	0.091	1.591	0.113
Residual	28	4526.396	0.801		
Total	33	5650.364	1		

Table S6. Results of linear regression in to test differences in alpha diversity indices Chao1, Shannon, and Simpson between human donor and FMT groups in 18S dataset. Pr(>|t|)<0.05 deemed as significant.

**Index**	**FMT group vs donor**	**Coef.**	**Std. Error**	**t value**	**Pr(>|t|)**
Chao	A	34.346	46.987	0.731	0.471
Chao	B	15.286	46.987	0.325	0.747
Chao	C	31.253	46.987	0.665	0.511
Chao	D	10.718	47.654	0.225	0.824
Chao	E	28.852	47.654	0.605	0.550
Chao	F	14.006	46.987	0.298	0.768
Shannon	A	0.733	0.631	1.162	0.255
Shannon	B	0.229	0.631	0.363	0.719
Shannon	C	0.234	0.631	0.371	0.714
Shannon	D	0.211	0.640	0.330	0.744
Shannon	E	0.344	0.640	0.538	0.595
Shannon	F	0.234	0.631	0.371	0.714
Simpson	A	0.306	0.254	1.204	0.239
Simpson	B	0.142	0.254	0.559	0.581
Simpson	C	0.148	0.254	0.585	0.563
Simpson	D	0.156	0.258	0.607	0.549
Simpson	E	0.188	0.258	0.731	0.471
Simpson	F	0.133	0.254	0.524	0.605

Table S7. Results of linear regression to test differences in alpha diversity indices Chao1, Shannon, and Simpson across different gut decontamination treatment, administration, and dosing interval in 18S dataset. Pr(>|t|)<0.05 deemed as significant.

Index	FMT condition	Coef.	Std. Error	t value	Pr(>|t|)
Chao	AB+T treatment	200.320	108.440	1847.000	0.074
Shannon	AB+T treatment	12027.000	0.398	3023.000	**0.005**
Simpson	AB+T treatment	0.238	0.090	2637.000	**0.013**
Chao	PO administration	-220.100	112.620	-1954.000	0.059
Shannon	PO administration	-0.254	0.469	-0.542	0.592
Simpson	PO administration	-0.021	0.104	-0.200	0.843
Chao	1 vs 3 (time/day)	-207.092	133.012	-1.557	0.130
Chao	1 vs 3x2 wk (time/day) dose	-233.113	133.012	-1.753	0.090
Chao	3x2 wk vs 3 (time/day) dose	-260.206	135.873	-0.192	0.849
Shannon	1 vs 3 (time/day) dose	-0.419	0.551	-0.761	0.453
Shannon	1 vs 3x2 wk (time/day) dose	-0.089	0.551	-0.162	0.873
Shannon	3x2 wk vs 3 (time/day) dose	0.330	0.563	0.586	0.562
Simpson	1 vs 3 (time/day) dose	-0.051	0.123	-0.420	0.677
Simpson	1 vs 3x2 wk (time/day) dose	0.010	0.123	0.081	0.936
Simpson	3x2 wk vs 3 (time/day) dose	0.061	0.125	0.490	0.627

Table S8. Differences in gut fungal community Aitchison distance between different FMT groups. Multilevel pairwise comparison performed and Bonferroni adjusted p-value<0.05 deemed as significant

FMT group pairs	Df	Sums Of Sqs	F Model	R2	p-value	p-adjusted
A vs B	1	150.312539	1.12235135	0.10090954	0.254	1.000
A vs C	1	304.875377	2.30236504	0.18714817	0.005	0.075
A vs D	1	410.484814	2.82922234	0.2391723	0.004	0.060
A vs E	1	486.550966	3.76565647	0.29498338	0.005	0.075
A vs F	1	413.866106	2.84593461	0.2215436	0.006	0.090
B vs C	1	202.863957	1.76044027	0.1496917	0.004	0.060
B vs D	1	184.978256	1.46814364	0.14024871	0.108	1.000
B vs E	1	329.609421	2.99333002	0.24958289	0.006	0.090
B vs F	1	257.689242	2.00942867	0.16732092	0.002	**0.030**
C vs D	1	93.4759899	0.75190439	0.07710334	0.925	1.000
C vs E	1	267.858015	2.47012728	0.21535308	0.014	0.210
C vs F	1	220.070864	1.73650661	0.14795771	0.026	0.390
D vs E	1	254.869096	2.12933307	0.21021454	0.03	0.450
D vs F	1	186.298538	1.34250559	0.12980468	0.249	1.000
E vs F	1	112.779862	0.91773577	0.09253481	0.491	1.000
